# The validity and reliability of the PHQ-9 in screening for post-stroke depression

**DOI:** 10.1186/s12888-020-02699-6

**Published:** 2020-06-09

**Authors:** Piyapat Dajpratham, Panate Pukrittayakamee, Wanlop Atsariyasing, Kamonporn Wannarit, Jariya Boonhong, Krit Pongpirul

**Affiliations:** 1 Department of Rehabilitation Medicine, Faculty of Medicine Siriraj Hospital, Mahidol University, 9th floor Srisangwal Building, Siriraj Hospital, Wanglang Road, Bangkok Noi, Bangkok, 10700 Thailand; 2 Department of Psychiatry, Faculty of Medicine Siriraj Hospital, Mahidol University, Bangkok, Thailand; 3 Department of Rehabilitation Medicine, Faculty of Medicine, Chulalongkorn University, Bangkok, Thailand; 4 Department of Preventive Medicine, Faculty of Medicine, Chulalongkorn University, Bangkok, Thailand

**Keywords:** Depression, Patient Health Questionnaire-9, Reliability, Screening, Stroke, Thai, Validity

## Abstract

**Background:**

Depression affects about 30% of stroke survivors within 5 years. Timely diagnosis and management of post-stroke depression facilitate motor recovery and improve independence. The original version of the Patient Health Questionnaire-9 (PHQ-9) is recognized as a good screening tool for post-stroke depression. However, no validation studies have been undertaken for the use of the Thai PHQ-9 in screening for depression among Thai stroke patients.

**Methods:**

The objectives were to determine the criterion validity and reliability of the Thai PHQ-9 in screening for post-stroke depression by comparing its results with those of a psychiatric interview as the gold standard. First-ever stroke patients aged ≥45 years with a stroke duration 2 weeks–2 years were administered the Thai PHQ-9. The gold standard was a psychiatric interview leading to a DSM-5 diagnosis of depressive disorder and adjustment disorder with a depressed mood. The summed-scored-based diagnosis of depression with the PHQ-9 was obtained. Validity and reliability analyses, and a receiver operating characteristic curve analysis, were performed.

**Results:**

In all, 115 stroke patients with a mean age of 64 years (SD: 10 years) were enrolled. The mean PHQ-9 score was 5.2 (SD: 4.8). Using the DSM-5 criteria, 11 patients (9.6%) were diagnosed with depressive disorder, 12 patients (10.5%) were diagnosed with adjustment disorder with a depressed mood. Both disorders were combined as a group of post-stroke depression. The Thai PHQ-9 had satisfactory internal consistency (Cronbach’s alpha: 0.78). The algorithm-based diagnosis of the Thai PHQ-9 had low sensitivity (0.52) but very high specificity (0.94) and positive likelihood ratio (9.6). Used as a summed-scored-based diagnosis, an optimal cut-off score of six revealed a sensitivity of 0.87, specificity of 0.75, positive predictive value of 0.46, negative predictive value of 0.95, and positive likelihood ratio of 3.5. The area under the curve was 0.87 (95% CI: 0.78–0.96).

**Conclusions:**

The Thai PHQ-9 has acceptable psychometric properties for detecting a mixture of major depression and adjustment disorder in post-stroke patients, with a recommended cut-off score of ≥6 for a Thai population.

## Background

Depression is the most common psychological problem experienced by survivors of a stroke [1]. The pool frequency is 31% of stroke survivors at any time up to 5 years after their stroke [2]. However, a review of prospective longitudinal research [3] showed that there is a biphasic pattern in post-stroke depression rates. The depressive symptoms gradually rise in the first 6 months, ease slightly at around 12 months, and worsen again during the second year after the stroke. Post-stroke depression (PSD) is associated with a longer length of hospital stay and decreased participation in rehabilitation programs, resulting in less functional improvement [4, 5]. After stroke patients are discharged, they tend to become physically inactive and socially isolated [6]. Depressed patients have fewer daily activities and a lower quality of life [7]. This may lead to more cognitive impairment [8] and increased mortality during the 2–5 years following the stroke [9].

It is difficult to make a diagnosis of depression after a stroke because the symptoms of depression can be confused with certain symptoms that are typical of stroke patients [10]. Screening for mood disorders after a stroke is recommended by many stroke and stroke-rehabilitation guidelines [11, 12]. Given that the availability of psychiatrists is limited in Thailand, there is a need for a screening tool to assist primary care physicians and other specialists in assessing for depression. Extensively studied in the non-Thai population and post-stroke patients, the Patient Health Questionnaire-9 (PHQ-9) has been reported to be a good PSD screening tool and to have the highest sensitivity [13, 14]. The PHQ-9 has also been translated into Thai (Thai PHQ-9) and validated in primary care patients [15]. The cut-off score of the Thai PHQ-9 for major depression in primary care patients is 9, which differs from the original version of the PHQ-9 [16]. As to PSD, Williams et al. [17] reported a cut-off score for the original version of 10 for the diagnosis of major depression, with a sensitivity of 91% and a specificity of 89%. However, the PHQ-9 has not yet been validated for PSD among Thais. Because Thailand and western countries have different health care systems, cultures, attitudes, mindsets, and family support systems, this study investigated the validity and reliability of the Thai PHQ-9 in screening for depression after stroke among Thais.

## Methods

### Subjects and procedures

Ethics approval was obtained from the Medical Ethics Committee of the Human Research Protection Unit, Faculty of Medicine Siriraj Hospital. The patients were recruited November 2017–December 2018 from the Department of Rehabilitation Medicine, Faculty of Medicine Siriraj Hospital, a tertiary hospital in Thailand. All patients gave written consent to participate. They were informed that their emotional status would be assessed via a questionnaire and a psychiatric interview. The patient inclusion criteria were aged ≥45 years; having a first-time stroke, as per WHO criteria [18], and with a stroke duration 2 weeks–2 years; stable vital signs, neurological signs, and stroke symptoms, as confirmed by a neurologist; and the ability to communicate in Thai. Excluded were patients with a cognitive impairment score of < 24, as measured by the Thai Mental State Examination [19], or a previous diagnosis of dementia, a psychiatric disorder, or another neurological disease.

Demographic characteristics were gathered from interviews with the enrolled patients, and information related to their stroke (such as any comorbid illnesses, and the types of stroke diagnosed from imaging studies) were obtained from medical records. The Modified Rankin Scales were also obtained to determine the level of disability of the participants. The Thai PHQ-9 [15] was administered by one of the researchers (PD) at either the inpatient rehabilitation ward or the outpatient rehabilitation clinic, depending on a patient’s visit. On the same day, a psychiatrist interviewed each patient in a private area and made a diagnosis according to the criteria detailed in the American Psychiatric Association’s Diagnostic and Statistical Manual of Mental Disorders, Fifth Edition (DSM-5). The researcher and the psychiatrist were blinded to each other’s assessment.

### Measures

#### Thai mental state Examination [19]

The Thai Mental State Examination (TMSE) is the first neuropsychiatric test that was used to provide a standard mental status examination of Thais. The maximum TMSE score is 30 points. For the diagnosis of a normal, healthy, older Thai person, a TMSE cut-off score of 24 points is used.

#### Modified Rankin Scale

The Modified Rankin Scale (MRS), a clinician-reported measure of global disability, has been widely applied to evaluate stroke recovery [20, 21]. It is an ordinal scale, with 7 categories ranging from zero (no symptoms) to six (death). The MRS assesses an individual’s ability to ambulate and complete the activities of daily living. MRS scores > 3 are defined as severe disability [22].

#### Thai PHQ-9 [15]

The PHQ-9 consists of 9 questions that are based on the 9 DSM-IV criteria for a major depressive disorder. The questionnaire explores the symptoms experienced by patients during the 2 immediately preceding weeks. The scores for each PHQ-9 item range from 0 (not at all), to 1 (several days), 2 (more than half of the days), and 3 (nearly every day). The PHQ-9 also provides a preliminary diagnosis of major depressive disorder using an algorithm-based diagnosis (≥ 5 items, including items 1 and/or 2, are rated ≥2), resulting in the total score for the questionnaire being 10 or higher. PHQ-9 can be used as a screening tool for the diagnosis of depression by using a summed-scored-based algorithm. The summed scores range from 0 to 27. Various cut-off scores allow for the determination of different degrees of depression. A study on the Thai PHQ-9 in the general Thai population reported that a summed score of 9 or greater signified a major depressive disorder, with a sensitivity of 0.84 and specificity of 0.77.

#### DSM-5

The DSM-5 criteria for depressive disorders and adjustment disorder were used as the reference standard [23]. A psychiatric interview was conducted for each patient. Three psychiatrists had a process of standardization whereby they discussed and agreed on the content of the interviews before they were conducted. Depressive disorders could be classified as a major depressive disorder, a persistent depressive disorder (dysthymia), a depressive disorder due to another medical condition, another specified depressive disorder, or as an unspecified depressive disorder. For adjustment disorder, the only adjustment disorder with depressed mood was selected as the symptoms of adjustment disorder with a depressed mood are similar to those of major depressive disorder [24].

### Data analysis

PASW Statistics for Windows, version 18.0 (SPSS Inc., Chicago, Ill., USA) [25] and MedCalc for Windows, version 15.0 (MedCalc Software, Ostend, Belgium) [26] were used for the statistical analyses. The demographic data, MRS, and PHQ-9 scores were analyzed by descriptive statistics. The quantitative data (age) was analyzed by an independent-sample t-test, while the stroke durations and Thai PHQ-9 scores were analyzed with the Mann–Whitney U test. Gender, education levels, risk factors, stroke pathology, side of weakness, and MRS scale were analyzed by Chi-square tests.

The stroke patients were divided into normal and depression groups, based on their psychiatric diagnoses. The psychiatrist determined the types of depressive disorders and adjustment disorder by using the relevant DSM-5 criteria. The depression scores of the normal and depression groups were analyzed by the independent-sample t-test. All analyses were significant at a p-value of < 0.05. Internal consistency was analyzed by Cronbach’s alpha. As a bivariate response, the psychiatric diagnosis of depression was used as the reference standard to calculate the sensitivities and specificities of all possible PHQ-9 cut-off scores. The positive and negative predictive values as well as the positive and negative likelihood ratios were calculated for each PHQ-9 cut-off score. Receiver-operator characteristic (ROC) analyses subsequently combined the instrument sensitivity and specificity into one measure (referred to as the area under the curve, or AUC) for all possible cut-off scores.

## Results

In all, 190 stroke patients were approached for participation. Seventy-five of those were excluded: 21 had recurrent stroke, 17 had cognitive impairment, 17 had aphasia, 10 were < 45 years, and 10 had a stroke duration > 2 years. After applying the exclusion criteria, 115 stroke patients were enrolled. They comprised 63 males (54.8%) and 52 females (45.2%), with a mean age of 64 years (SD: 10 years; min, max: 45, 88). The majority had graduated primary school, followed by lower-secondary school and upper-secondary school. The comorbid illnesses found were, in descending order of frequency, hypertension, dyslipidemia, diabetes mellitus, and heart disease. The median duration of stroke was 59 days. The large majority of patients (81.7%) suffered from ischemic stroke, and left-side weakness was dominant (61%). Most patients (65.2%) were recruited from inpatient rehabilitation.

All patients were administered the PHQ-9 as the index test. The reference standard was the psychiatric interview conducted on the same day, with the resultant diagnosis based on the DSM-5 criteria. The psychiatrist who administered the interview was blinded to the corresponding score for the index test, and all interviews were conducted regardless of the index test scores. The mean Thai PHQ-9 score was 5.2 ± 4.8. According to the DSM-5 criteria, 11 patients (9.6%) were diagnosed with depressive disorder, 12 patients (10.5%) were diagnosed as adjustment disorder with a depressed mood and the rest of 92 patients (80%) were normal. In the depressive disorder group, eight (6.9%) were classified as having a major depressive disorder (MDD), two (1.7%) with an unspecified depressive disorder, and one (0.9%) with another specified depressive disorder. Although the number and quality of symptoms of adjustment disorder with a depressed mood are less than those of major depressive disorder, [24] this study combined the depressive disorder group with the adjustment disorder group and named it as a depression group for the analysis.

The demographic characteristics of the normal and depression groups revealed no statistically significant differences (Table 1). However, the MRS and the median PHQ-9 scores of the groups differed. MRS scores of 0–3 were defined as no-severe disability, while MRS scores > 3 were defined as severe disability; more stroke patients were disabled in the depression group (78%) than in the normal group (55.4%).

**Table 1:** The baseline characteristics of the stroke patients

Variables	Normal (*N* = 92)	PSD (*N* = 23)	*P*-value
*Demographic-related*			
Age^a^	64.7 (9.5)	64.6 (12.2)	0.960
Gender^b^			0.092
• Male	54 (58.7)	9 (39.1)	
• Female	38 (41.3)	14 (60.9)	
Education level^b^			0.430
• Primary school	42 (45.7)	13 (56.6)	
• Secondary school	26 (28.3)	5 (21.7)	
• Bachelor degree and higher	24 (26.0)	5 (21.7)	
Comorbid illness^b^			
• Hypertension	77 (83.7)	21 (91.3)	0.518
• Dyslipidemia	53 (57.6)	17 (73.9)	0.152
• Diabetes mellitus	37 (40.2)	12 (52.2)	0.300
• Smoking	21 (22.8)	4 (17.4)	0.572
• Heart disease	19 (20.7)	6 (26.1)	0.572
Duration of stroke^b^			0.293
• ≤ 3 months	58 (63.0)	16 (69.6)	
• 3–6 months	14 (15.2)	5 (21.7)	
• > 6 months	20 (21.7)	2 (8.7)	
Pathology of stroke^b^			0.561
• Infarction	74 (80.4)	20 (87.0)	
• Hemorrhage	18 (19.6)	3 (13.0)	
Side of weakness^b^			0.339
• Left	54 (58.7)	16 (69.6)	
• Right	38 (41.3)	7 (30.4)	
Setting^b^			0.793
• Inpatient	60 (65.2)	15 (65.2)	
• Outpatient	32 (34.8)	8 (34.8)	
*Disability-related*			
Modified Rankin Scale^b^			0.036*
• 1	7 (7.6)	2 (8.7)	
• 2	16 (17.4)	0 (0.0)	
• 3	18 (19.6)	3 (13.0)	
• 4	50 (54.3)	15 (65.2)	
• 5	1 (1.1)	3 (13.0)	
*Depression-related*			
Median PHQ-9 score^c^	4.0 (0.5, 5.75)	10.0 (7.0, 15.0)	< 0.001*

^a^ Mean (SD)^b^; number (%)^c^; median (IQR 25,75), *significant at *p*-value < 0.05

### Reliability and item analysis

As presented in Table 2, the highest mean score of the nine PHQ-9 items was found for Item 3 (“trouble falling or staying asleep, or sleeping too much”). Item 9 (“thoughts that you would be better off dead or of hurting yourself”) had the lowest score. As to the internal consistency of the PHQ-9, Cronbach’s alpha was 0.78. All items, if deleted, would consistently decrease the total scale alpha. The least item-total correlation was for Item 5 (“poor appetite or overeating”).

**Table 2:** Mean score, standard deviation, and internal reliability score for each PHQ-9 score

PHQ-9 items	Mean	Standard deviation	Corrected item-total correlation	Cronbach’s alpha if item deleted
1. Little interest or pleasure in doing things	0.72	0.881	0.612	0.708
2. Feeling down, depressed, or hopeless	0.64	0.926	0.516	0.723
3. Trouble falling or staying asleep, or sleeping too much	1.11	1.256	0.404	0.749
4. Feeling tired or having little energy	0.68	0.984	0.321	0.755
5. Poor appetite or overeating	0.47	0.955	0.199	0.773
6. Feeling bad about yourself – or that you are a failure	0.71	1.015	0.612	0.704
7. Trouble concentrating on things	0.27	0.641	0.345	0.749
8. Moving or speaking so slowly that other people have noticed	0.35	0.731	0.555	0.722
9. Thoughts that you would be better off dead or of hurting yourself	0.25	0.662	0.525	0.729

### Validity analysis

A comparison was made of the performance of the Thai PHQ-9 against the diagnosis of depressive and adjustment disorders (based on the DSM-5 criteria for depressive disorders and adjustment disorder with a depressed mood as the standard). According to the DSM-5 criteria, 11 patients (9.6%) met the diagnosis of depressive disorder and 12 patients (10.5%) met the diagnosis of adjustment disorder with a depressed mood. These two disorders were combined as a depression group. The median Thai PHQ-9 score for the depression group was 10 (IQR 25, 75%: 7, 15) whereas the median score of the normal group was 4 (IQR 25, 75%: 0.5, 5.75). The differences in the median PHQ-9 scores of the 2 groups were statistically significant.

**Fig. 1:**
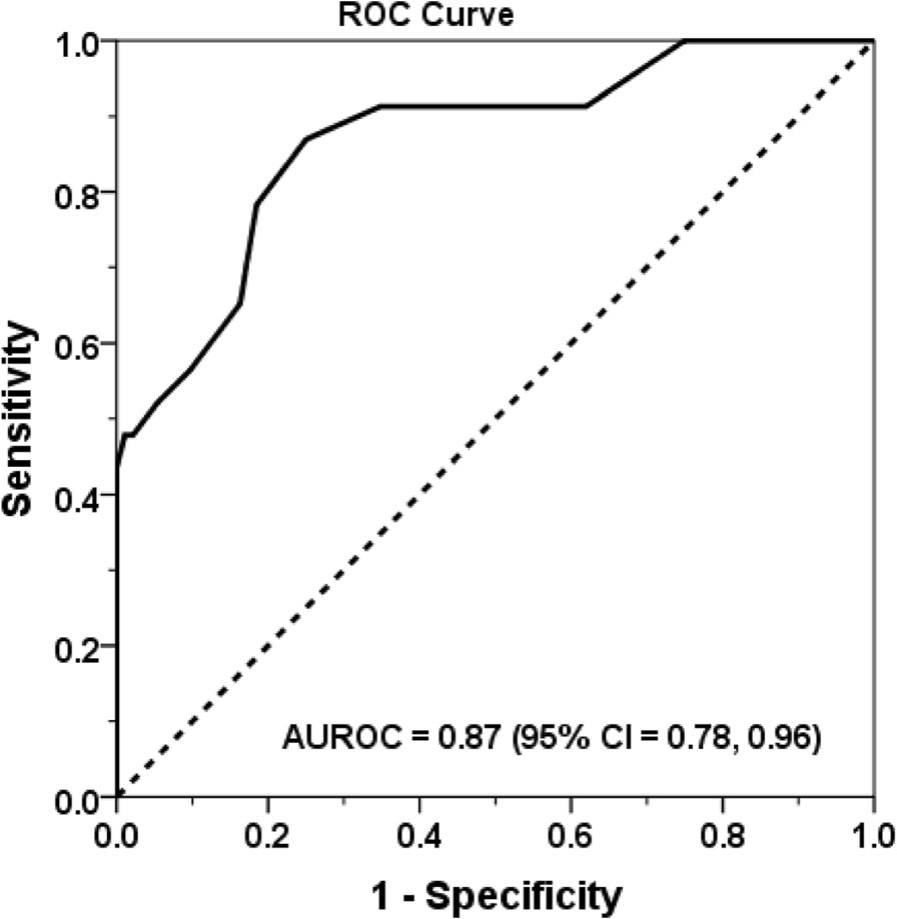
The receiver operating characteristic curve of the PHQ-9 for screening depression among stroke patients

When using the algorithm-based diagnosis, an assessment of the validity of the Thai PHQ-9 index test revealed a sensitivity of 34.8%, specificity of 97.8%, positive predictive value of 80%, negative predictive value of 85.7%, and positive likelihood ratio of 16.0 (Table 3). As to using the summed-scored-based diagnosis, the corresponding values for different PHQ-9 thresholds in diagnosing PSD are detailed in Table 2. The cut-off score of 6 showed the highest Youden’s index. This cut-off score had a sensitivity of 87.0% (95% CI: 66.4, 97.2), specificity of 75.0% (95% CI: 64.9, 83.4), positive predictive value of 46.5% (95% CI: 37.1, 56.2), negative predictive value of 95.8% (95% CI: 88.8, 98.5), positive likelihood ratio of 3.5 (95% CI: 2.4, 5.1), and negative likelihood ratio of 0.2 (95% CI: 0.1, 0.5). The ROC curve illustrates that the PHQ-9 performed well in identifying patients with PSD (Fig. 1). The AUC in our study was 0.87 (95% CI: 0.78, 0.96), which represents good discrimination.

**Table 3:** The performance of different PHQ-9 cut-off scores in detecting depression

Score	Sensitivity (%) (95% CI)	Specificity (%) (95% CI)	Positive predictive value (%) (95% CI)	Negative predictive value (%) (95% CI)	Positive likelihood ratio (95% CI)	Negative likelihood ratio (95% CI)	Accuracy (95% CI)	Youden’s index
The algorithm-based diagnosis								
≥ 10	34.8 (16.4, 57.3)	97.8 (92.4, 99.7)	80.0 (47.6, 94.6)	85.7 (81.6, 89.0)	16.0 (3.6, 70.3)	85.7 (81.6, 89.0)	85.2 (77.4, 91.2)	–
The summed-item-based diagnosis								
≥ 5	91.3 (71.9, 98.9)	65.2 (54.6, 74.8)	39.6 (32.6, 47.2)	96.8 (88.8, 99.1)	2.62 (1.9, 3.6)	0.13 (0.04, 0.5)	70.4 (61.2, 78.6)	0.565
≥ 6	87.0 (66.4, 97.2)	75.0 (64.9, 83.4)	46.5 (37.1, 56.2)	95.8 (88.8, 98.5)	3.5 (2.4, 5.1)	0.2 (0.1, 0.5)	77.4 (68.6, 84.7)	0.620
≥ 7	78.3 (56.3, 92.5)	81.5 (72.1, 88.8)	51.4 (39.6, 63.1)	93.8 (87.3, 97.0)	4.2 (2.6, 6.8)	0.3 (0.1, 0.6)	80.9 (72.5, 87.6)	0.598
≥ 8	65.2 (42.7, 83.6)	83.7 (74.5, 90.6)	50.0 (36.6, 63.4)	90.6 (84.5, 94.4)	4.0 (2.3, 6.9)	0.42 (0.2, 0.7)	80.0 (71.5, 86.9)	0.489
≥ 9	56.5 (34.5, 76.8)	90.2 (82.2, 95.4)	59.1 (41.4, 74.7)	89.3 (83.8, 93.0)	5.8 (2.8, 11.8)	0.5 (0.3, 0.8)	83.5 (75.4, 89.7)	0.467
≥ 10	52.2 (30.59, 73.2)	94.6 (87.7, 98.2)	70.6 (48.4, 85.9)	88.8 (83.7, 92.4)	9.6 (3.7, 24.5)	0.5 (0.3, 0.8)	86.1 (78.4, 91.8)	0.467

## Discussion

This study was the first in Thailand to determine the validity of a depression screening questionnaire with stroke patients. The questionnaire investigated was the PHQ-9, one of the good screening tools for PSD [14]. The reference standard was a psychiatric interview based on the DSM-5 criteria for depressive disorders and adjustment disorders. In this study, adjustment disorder with a depressed mood was also diagnosed in a number of patients. Casey et al. reported that it was difficult to identify distinguishing features between adjustment disorder from the depressive episode [24]. After stroke, abrupt physical impairment leads to physical disability and this is an immense and ongoing stressor for a stroke survivor. Although physical rehabilitation can attenuate the impairment, the patients need to actively participate the rehabilitation program. Low mood, tearfulness, or feelings of hopelessness are predominant depressive symptoms in adjustment disorder with a depressed mood [27]. These symptoms would lessen motivation to achieve rehabilitation training goal. Therefore, the stroke patients with adjustment disorder pose a risk to have poor progression in rehabilitation training. In order to make use of the questionnaire screening for depression after stroke, both disorders were combined as a group of depression. The validity of the PHQ-9 in screening for depression was good in terms of its discriminatory power (AUC: 0.87) relative to the gold-standard, DSM-5 criteria. In addition, its internal consistency was acceptable (Cronbach’s alpha: 0.78).

Depressive disorder was found in 11 patients (9.6%), which was less than the corresponding figures reported by other studies. A meta-analysis conducted by Hackett and Pickles [2] found that 31% of stroke patients developed depression or depressive symptoms in any setting and at any time up to 5 years following their stroke. Robinson [28] undertook a pooled analysis and reported mean incidences for major and minor depression of 19.3 and 18.5%, respectively, among hospitalized patients in acute care and rehabilitation hospitals. By comparison, the low incidence in the present study probably stemmed from having the criterion that only stroke patients aged ≥45 years would be included. Previous research has found that younger stroke survivors are more likely to become depressed than older survivors [29, 30]. Nevertheless, the incidence established by the current study is in line with that of research by Fuentes et al., which recruited stroke patients of the same age group and found a low depression incidence of 9.9% [31]. In this study, adjustment disorder with a depressed mood were diagnosed in 12 patients (10.5%). The pooled prevalence of adjustment disorder after stroke across all settings was 6.9% [32] which was lower than the number found in this study. Most of the stroke patients (69.6%) recruited to the study had duration within 3 months and this could contribute to the higher prevalence of adjustment disorder.

Moving on to the demographic characteristics of stroke patients with and without PSD, our study revealed no significant differences in the demographic-related variables of the groups. In the case of the disability-related variable, the MRS was used to determine the level of disability after stroke. The patients with an MRS score > 3, who were classified as having a severe disability, appeared more frequently in the depression group. PSD has been found to be associated with more severe neurological deficits and physical disabilities in the acute and chronic phases [33, 34].

The internal consistency of the Thai PHQ-9 administered to the stroke patients in this study was 0.78, which is considered acceptable. However, the level of internal consistency we found differed from that of the original version of the PHQ-9. The original studies—performed in primary care and in obstetrics and gynecology settings—showed an internal consistency of 0.89 and 0.86, respectively [16]. In addition, Turner et al., who utilized PHQ-9 to screen for PSD, found an internal consistency of 0.82 [13]. In the case of the Thai version of the PHQ-9, a validity study on the Thai population reported an internal consistency of 0.79 [15]. Later, Lee and Dajpratham, who employed the Thai version on elderly Thais, reported an internal consistency of 0.76 [35]. In the current research, the internal consistency was 0.78, which means that it is highly congruent with those two earlier studies using the Thai version of the PHQ-9.

The Thai PHQ-9 can be used as a screening tool since the AUC showed a good level of discriminatory power (AUC: 0.87). The results of our study are in line with several other investigations that have reported a good discriminatory power for the PHQ-9, with an AUC of > 0.8 [13, 17, 36–38]. As to its validity, the PHQ-9 score can be used in 2 ways to diagnose depression. The first is an algorithm-based diagnosis for major depression, with a cut-off score of 10. In 2015, Manea et al. [39] conducted a diagnosis meta-analysis of the PHQ-9 algorithm-based scoring method as a screening tool for depression. They found that although the sensitivity was as low as 53% (95% CI: 42–65), the specificity was as high as 94% (95% CI: 91–96). Our study applied the algorithm-based diagnosis for PSD in a tertiary-hospital setting. Our evaluation of the diagnostic accuracy revealed low sensitivity and high specificity (Table 2), consistent with the results of the work by Manea et al. [39] Low sensitivity is not a good property of a screening tool. Therefore, all of the previous PHQ-9 validation studies for the detection of PSD have used the alternative diagnostic approach, summed-scored-based diagnosis, for their comparisons with various structured interviews as their reference standard [13, 17, 36, 38, 40]. Pettersson et al. [41] performed a systematic review to explore the diagnostic accuracy of the structured interviews as index tests. The only structured interviews which were found to have sufficient accuracy for the diagnosis of depression disorders were the Structured Clinical Interview for DSM-IV (SCID) and the Mini International Neuropsychiatric Interview (MINI). The summed-scored-based PHQ-9 diagnoses in the current research were validated against the psychiatric interviews that were based on DSM-5 criteria. Our analysis revealed an optimum cut-off score of 6 for the diagnosis of depression. This finding differed from those of other studies [13, 17]. Turner et al. [13] validated the PHQ-9 for the detection of PSD against the DSM-IV criteria; they reported a summed score greater than 8 as the cut-off score for diagnosis. Similarly, Williams et al. [17] reported a summed score of 10 or greater as the cut-off score for diagnosis.

The suggestion for further study is to expand the sample size so the prevalence of depressive disorder after stroke would be enough to provide the specific entity for the depression screening tool. There were some limitations to this study. Firstly, the high mean age of the participants, 64 years, meant that the findings cannot be generalized to younger stroke patients. However, the incidence of stroke at a younger age is lower and only represents a small proportion in clinical practice. Secondly, only participants who could communicate were recruited. Stroke patients who are unable to communicate would probably be very depressed. Moreover, the mood assessment scale for patients who cannot communicate is different. Thirdly, this study did not include other psychiatric disorders after stroke. Finally, this study did not perform test–retest reliability; consequently, the temporal stability of the measure for Thai people with a stroke is presently unknown.

## Conclusions

The Thai version of the PHQ-9 had acceptable properties for detecting a mixture of major depression and adjustment disorder in post-stroke patients. The summed-scored-based depression diagnosis should therefore be employed for screening, with a cut-off score of 6 signifying PSD.

## Data Availability

All data generated or analyzed during this study are included in this published article and its supplementary information files.

## Abbreviations

AUC: Area under the curve
MINI: Mini International Neuropsychiatric Interview
PHQ-9: Patient Health Questionnaire 9
PSD: Post-stroke depression
ROC: Receiver operating characteristic curve
SCID: Structured Clinical Interview for DSM-IV
